# Distribution of FIB-4 index in the general population: analysis of 75,666 residents who underwent health checkups

**DOI:** 10.1186/s12876-022-02290-1

**Published:** 2022-05-13

**Authors:** Aya Sugiyama, Akemi Kurisu, Bunthen E, Serge Ouoba, Ko Ko, Anvarjon Rakhimov, Tomoyuki Akita, Takayuki Harakawa, Toru Sako, Makoto Koshiyama, Takashi Kumada, Junko Tanaka

**Affiliations:** 1 Department of Epidemiology, Infectious Disease Control and Prevention, Graduate School of Biomedical and Health Sciences, Hiroshima University, 1-2-3, Kasumi, Minami-ku, Hiroshima, 734-8551 Japan; 2 Payment Certification Agency (PCA), Ministry of Health, Phnom Penh, Cambodia; 3 Unite De Recherche Clinique De Nanoro (URCN), Institut de Recherche en Science de La Sante (IRSS), Nanoro, Burkina Faso; 4 General Affairs, Foundation for Community Health and Medicine Promotion in Hiroshima Prefecture, Hiroshima, Japan; 5 Iwate Prefectural Preventive Medicine Association, Iwate, Japan; 6 Department of Nursing, Faculty of Nursing, Gifu Kyoritsu University, Ogaki, Gifu Japan

**Keywords:** Fatty liver, Non-alcoholic fatty liver disease, Liver fibrosis, Indirect biomarker, Scoring system, Ultrasonography, Age effect, Aspartate aminotransferase, Alanine aminotransferase, Japan

## Abstract

**Background:**

Fatty liver is frequently found in a general population, and it is critical to detect advanced fibrosis. FIB-4 index is considered a useful marker for evaluating liver fibrosis but the distribution of FIB-4 index in the general population remains unknown.

**Methods:**

This cross-sectional study included residents who underwent ultrasonography at health checkups in Hiroshima or Iwate prefectures. The distribution of FIB-4 index in the total study population (N = 75,666) as well as in non-alcoholic fatty liver disease (NAFLD) populations (N = 17,968) and non-drinkers without fatty liver populations (N = 47,222) was evaluated. The distribution of aspartate aminotransferase (AST) levels, alanine aminotransferase (ALT) levels was also evaluated.

**Results:**

The mean FIB-4 index in the total study population was 1.20 ± 0.63. FIB-4 index ≥ 2.67, which indicates a high risk of liver fibrosis, was found in 16.4% of those aged ≥ 70 years. In the NAFLD population, 58.1% of those in their 60 s and 88.1% of those ≥ 70 years met the criteria for referral to hepatologists by using the recommended FIB-4 index cutoff value (≥ 1.3). The mean FIB-4 index in the NAFLD population (1.12 ± 0.58) was significantly lower than in the non-drinkers without fatty liver (1.23 ± 0.63, *p* < 0.0001). The non-drinkers without fatty liver tended to have higher AST relative to ALT levels (60.0% with AST/ALT > 1.0), whereas the results in the NAFLD population were opposite (14.8% with AST/ALT > 1.0). AST > ALT resulted in a higher FIB-4 index in non-drinkers without fatty liver due to the nature of FIB-4 index formula.

**Conclusions:**

The cutoff value of FIB-4 index (≥ 1.3) for triaging the elderly people with fatty liver for referral to hepatologists should be reconsidered to avoid over-referral. Due to the impact of age and characteristics of AST/ALT ratios, there is no prospect of using FIB-4 index for primary screening for liver fibrosis in a general population of unknown presence or absence of liver disease, even though it can be easily calculated using routine clinical indices. It is desired to develop a non-invasive method for picking up cases with advanced fibrosis latent in the general population.

**Supplementary Information:**

The online version contains supplementary material available at 10.1186/s12876-022-02290-1.

## Background

Non-alcoholic fatty liver disease (NAFLD) is one of the most common diseases found by abdominal ultrasonography at health checkups. Of the 75,670 residents in Japan who underwent abdominal ultrasonography at health checkups, 23.7% were diagnosed with NAFLD [1]. NAFLD is categorized into non-alcoholic fatty liver (NAFL), which typically does not progress, and non-alcoholic steatohepatitis (NASH), which can progress to cirrhosis and liver cancer. Liver fibrosis is the most important factor associated with prognosis in patients with NAFLD [2]. It is critical to find cases with advanced fibrosis in NAFLD. Although histological assessment with liver biopsy is essential to distinguish NAFL from NASH and determine the liver fibrosis stage, it cannot be performed in all patients with fatty liver. Therefore, the use of clinical scoring tools such as the fibrosis-4 (FIB-4) index has been recommended to non-invasively detect advanced liver fibrosis [3–5].

FIB-4 index is based on aspartate aminotransferase (AST) level, alanine aminotransferase (ALT) level, platelet count, and age. When evaluating liver fibrosis in patients with NAFLD, a FIB-4 index < 1.3 is categorized as low risk, while a FIB-4 index ≥ 2.67 is categorized as high risk of fibrosis [3, 6]. The recommendation for referral from primary care clinicians to hepatologists is a FIB-4 index ≥ 1.3 [3, 7]. However, there is a paucity of data on the distribution of FIB-4 index in the general population with fatty liver detected by ultrasonography at health checkups in Japan. In addition, since FIB-4 index can be easily calculated using routine clinical and biochemical indices, it may be useful if it can be used for primary screening for advanced fibrosis in the general population. However, the characteristics of FIB-4 index distribution in the general population remain unknown [8]. This large-scale community-based cross-sectional study was conducted to present data on the distribution of FIB-4 index in a general population cohort in Japan.

## Patients and methods

**Fig. 1:**
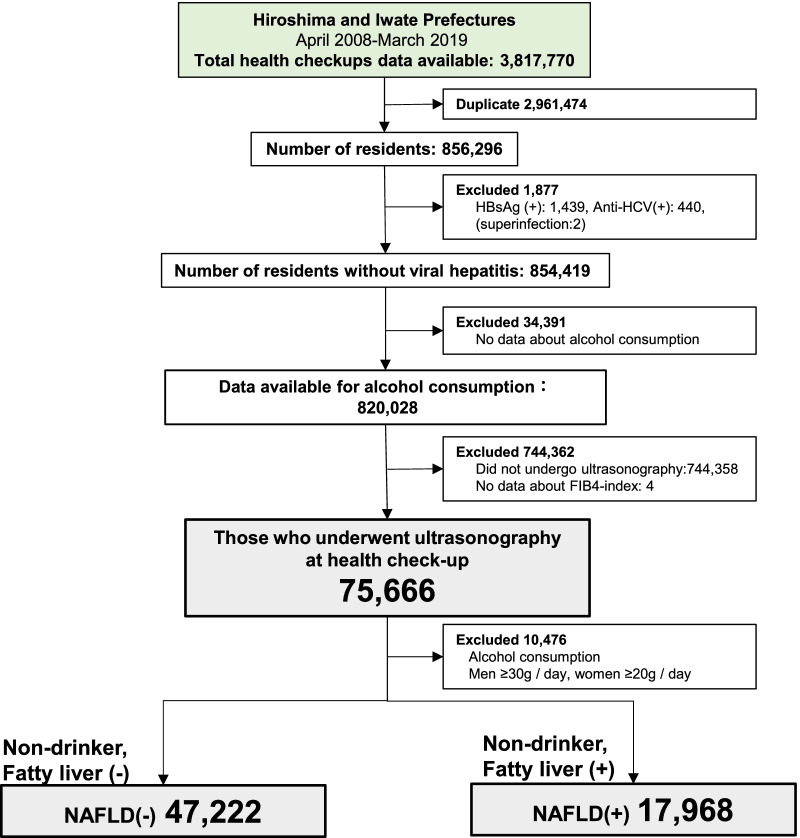
Procedure for selecting study subjects. Flowchart of selecting study subjects from the health checkup database in Hiroshima (April 2013–July 2018) and Iwate (April 2008–March 2019) prefectures. A total of 75,666 individuals without HBV or HCV provided blood samples, answers about alcohol consumption, and underwent ultrasonography for health checkups, and 17,968 residents were diagnosed as NAFLD. *HBsAg* hepatitis B surface antigen, *Anti-HCV* anti hepatitis C virus antibody, *HBV* hepatitis B virus, *HCV* hepatitis C virus, *NAFLD* non-alcoholic fatty liver disease

This study was designed as a cross-sectional study. Study participants underwent ultrasonography at health checkups in Hiroshima Prefecture or Iwate Prefecture. Data from these participants were extracted from anonymized data of all residents who underwent health checkups provided by the Foundation for Community Health and Medicine Promotion in Hiroshima between April 2013 and July 2018 (5 years) (total number of individuals = 172,819; number of individuals excluding duplicates = 58,652) and the Iwate Prefectural Preventive Medicine Association between April 2008 and March 2019 (11 years) (total number of individuals = 3,644,951; number of individuals excluding duplicates = 797,644). After excluding duplicates, 856,296 individuals in Hiroshima and Iwate who received a health checkup during the study period were screened to determine if they met study inclusion criteria. Next, 1877 patients with hepatitis B virus (HBV) or hepatitis C virus (HCV) infections were excluded. Of the remaining 854,419 individuals, 75,666 (44,420 males and 31,246 females) who underwent ultrasonography at health checkups, completed an alcohol use questionnaire, and had laboratory testing results were included in the analysis (Fig. 1). For this study, NAFLD was defined as fatty liver diagnosed with abdominal ultrasonography in those who did not test positive for HBV or HCV and did not consume more than 30 g/day of alcohol for men and 20 g/day for women based on the Japanese guidelines [3]. Fatty liver was diagnosed if any of the following four ultrasonic findings were present: bright liver, hepato-renal contrast, deep attenuation, and vascular blurring [9]. A clinical laboratory technician made the primary diagnosis, and a radiologist reviewed the images to make the final diagnosis. Based on this definition, 17,968 (23.7%) individuals who underwent ultrasonography were determined to have NAFLD. Non-drinkers without fatty liver by ultrasonography were 47,222 (Fig. 1). FIB-4 index distribution (mean ± standard deviation, SD) at the time of first ultrasonography during the study period was calculated in the total population who received ultrasonography (N = 75,666), the NAFLD population (N = 17,968), and the non-drinkers without fatty liver population (N = 47,222) using the formula below:

$$\mathrm{FIB-4 index} =\frac{\mathrm{AST }(\mathrm{IU}/\mathrm{L})\times \mathrm{Age}(\mathrm{years})}{\mathrm{Platelet count }({10}^{9}/\mathrm{L})\times \sqrt{\mathrm{ALT}(\mathrm{IU}/\mathrm{L})}}$$

After standardizing the number of age-specific populations, the proportion of age groups in each FIB-4 index value in the total population who received ultrasonography (N = 75,666) was also calculated.

In addition, since FIB-4 index formula includes AST and ALT, we evaluated the distribution of AST levels, ALT levels, and AST/ALT ratios (AAR). As for the data of Iwate, only the aggregated value could be obtained according to the facility rule. Therefore, only the Hiroshima data, consisting of 5999 residents who received ultrasonography, including 1399 with NAFLD, and 3781 non-drinkers without fatty liver, were used for this analysis.

### Statistical analysis

Differences between two groups were evaluated using the Wilcoxon test for continuous variables and the Chi-square test for categorical variables. The proportions of FIB-4 index ≥ 2.67, the cutoff value for high risk of liver fibrosis, were compared by age group in both non-drinkers without fatty liver and NAFLD groups using the Cochran-Armitage test. The correlations between AST and ALT values in both groups were analyzed using the Pearson correlation method. Statistical analyses were conducted using JMP14.2.0 software (SAS Institute Inc., Cary, NC, USA), and *p* values less than 0.05 were regarded as statistically significant.

## Results

**Fig. 2:**
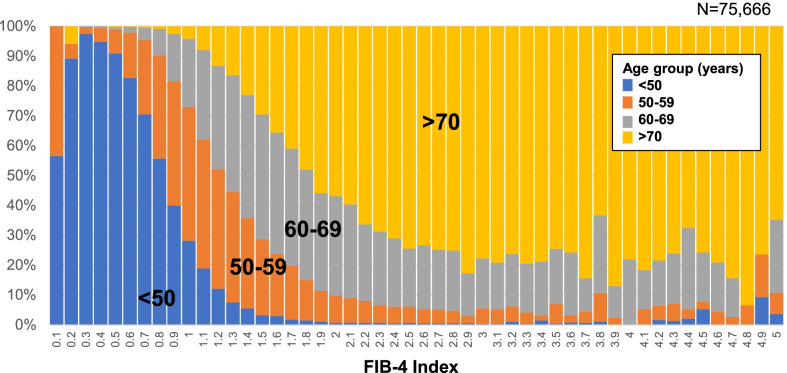
Proportion of age group in each FIB-4 index value in residents who underwent abdominal ultrasonography at health checkups in Japan (N = 75,666). This figure shows the proportion of age group in each FIB-4 index value in residents who underwent ultrasonography at health checkups in Japan. Blue color represents the age group under 50, orange represents the age group 50–59, gray represents the age group 60–69, and yellow represents the age group over 70. Most of the high FIB-4 values are in the 70 + age group

The mean ± SD of FIB-4 index in the total population who received ultrasonography at health checkups (N = 75,666) was 1.20 ± 0.63. FIB-4 index was higher in older groups: 0.82 ± 0.31 in those aged < 50 years, 1.23 ± 0.44 in those aged 50–59 years, 1.60 ± 0.75 in those aged 60–69 years, and 2.10 ± 0.75 in those aged ≥ 70 years (Table 1). FIB-4–index ≥ 2.67, which is the criterion for high risk of liver fibrosis, was found in 3.8% of those aged 60–69 years and 16.4% of those aged ≥ 70 years old in the total population (Table 1). The proportion of FIB-4 index ≥ 2.67 was significantly higher in the elderly age group using the Cochran-Armitage test (*p* < 0.0001). The proportion of age group in each FIB-4 Index value is shown in Fig. 2. Among those with a FIB-4 index value ≥ 2.67, 70% or more were 70 years or older. There was no significant difference in the distribution of FIB4-index by sex (*p* = 0.2200), as shown in Additional file 1: Fig. S1.

**Table 1:** Distribution of FIB-4 index in those who received abdominal ultrasonography at health checkups in Japan

	Age (years)	N	FIB-4 index					
			Mean ± SD	Median	Min–Max	FIB4-index < 1.30 (%)	FIB4-index ≥ 2.67 (%)	Cochran-Armitage test for the proportion of FIB-4 index ≥ 2.67 by age group
Total population who received ultrasonography (N = 75,666)	< 50	32,103	0.82 ± 0.31	0.62	0.18–12.07	93.3	0.2	*p* < 0.0001
	50–59	20,868	1.23 ± 0.44	1.16	0.17–15.39	68.9	0.8	
	60–69	16,854	1.60 ± 0.66	1.50	0.34–32.48	31.0	3.8	
	≥ 70	5841	2.10 ± 0.75	1.97	0.23–15.40	7.8	16.4	
	Overall	75,666	1.20 ± 0.63	1.07	0.17–32.48	65.3	2.3	
Non-drinkers without fatty liver (N = 47,222)	< 50	20,133	0.82 ± 0.28	0.78	0.21–6.64	94.9	0.1	*p* < 0.0001
	50–59	11,946	1.24 ± 0.40	1.18	0.22–5.97	62.3	0.7	
	60–69	10,649	1.61 ± 0.63	1.53	0.44–32.48	27.9	3.4	
	≥ 70	4494	2.13 ± 0.75	2.01	0.23–15.40	6.8	17.3	
	Overall	47,222	1.23 ± 0.63	1.10	0.21–32.48	63.2	2.3	
Non-drinkers with fatty liver (NAFLD) (N = 17,968)	< 50	6934	0.75 ± 0.31	0.71	0.18–12.07	96.4	0.1	*p* = 0.0008
	50–59	5559	1.13 ± 0.38	1.06	0.33–4.34	73.7	0.5	
	60–69	4350	1.48 ± 0.66	1.38	0.34–26.70	41.9	2.7	
	≥ 70	1125	1.92 ± 0.66	1.82	0.58–7.02	11.9	10.3	
	Overall	17,968	1.12 ± 0.58	1.01	0.18–26.70	70.9	1.6	

*SD* standard deviation, *NAFLD* non-alcoholic fatty liver disease

The proportion of FIB-4 index ≥ 2.67, high risk of liver fibrosis, were compared by age group by the Cochran-Armitage test

**Fig. 3:**
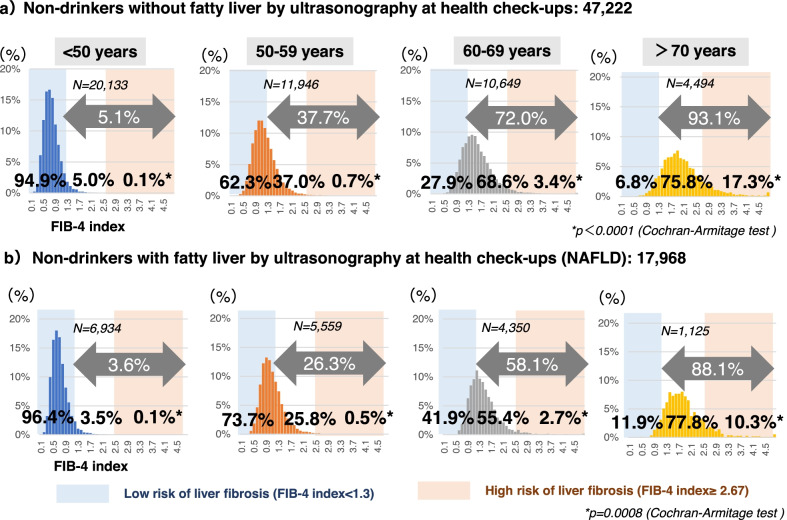
Distribution of FIB4 index values by age in non-drinkers with or without fatty liver. This figure shows the distribution of FIB-4 index by age group in **a** non-drinkers without fatty liver by ultrasonography at health checkups (N = 47,222) and **b** non-drinkers with fatty liver by ultrasonography at health checkups (NAFLD) (N = 17,968). Light pink color areas represent high risk of liver fibrosis defined by FIB-4 index ≥ 2.67. Light blue color areas represent low-risk of liver fibrosis defined by FIB-4 index < 1.3. The proportions of FIB-4 index ≥ 2.67, the cutoff for high risk of liver fibrosis, were compared by age group in both non-drinkers without fatty liver and NAFLD groups using the Cochran-Armitage test. *NAFLD* non-alcoholic fatty liver disease

Among the NAFLD population (N = 17,968), FIB-4–index < 1.3, which is the criterion for low risk of liver fibrosis, was found in 96.4% of those aged < 50 years, 73.7% of those aged 50–59 years old, 41.9% of those aged 60–69 years old, and 11.9% of those aged ≥ 70 years old. FIB-4 index tended to be higher in older populations in both groups with and without NAFLD (*p* < 0.0001, *p* = 0.0008 respectively, Cochran-Armitage Test) (Table 1, Fig. 3). The mean FIB-4 index in the NAFLD population (N = 17,968) was 1.12 ± 0.58, which is significantly lower than in the non-drinkers without fatty liver (N = 47,222, 1.23 ± 0.63, *p* < 0.0001, Wilcoxon test) (Additional file 2: Table S1). Comparing by age group, FIB-4 index value was significantly lower in the NAFLD population than in the non-drinkers without fatty liver in all age groups (*p* < 0.0001, respectively, Wilcoxon test) (Additional file 2: Table S1).

**Fig. 4:**
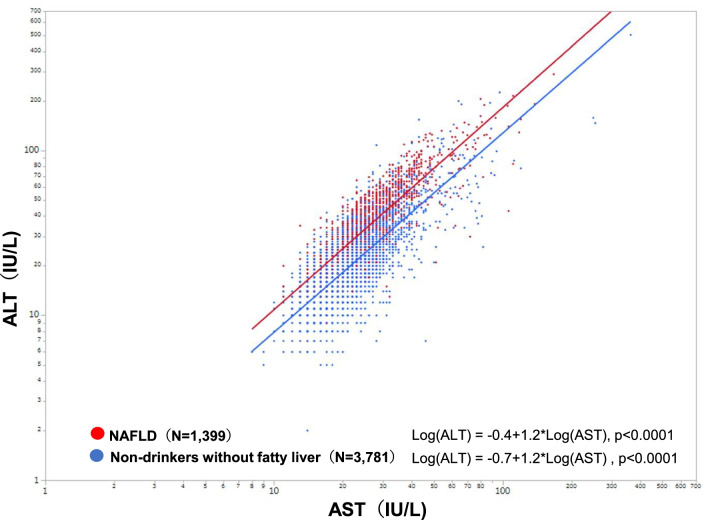
Distribution of AST and ALT values in 5,180 non-drinkers undergoing abdominal ultrasonography at health checkups in Hiroshima. The correlations between AST and ALT values in non-drinkers without fatty liver and NAFLD groups were analyzed using the Pearson correlation method**.** Red dots represent non-drinkers with fatty liver (NAFLD) (N = 1399). Blue dots represent non-drinkers without fatty liver (N = 3781) *AST* alanine aminotransferase, *ALT* aspartate aminotransferase, *NAFLD* non-alcoholic fatty liver disease

The mean AST level, ALT level, and AAR among those with NAFLD (N = 1399, Hiroshima) were 28.0 ± 13.6 IU/L, 40.7 ± 26.2 IU/L, and 0.78 ± 0.26, respectively. Among the non-drinkers without fatty liver (N = 3781, Hiroshima), these values were 20.9 ± 9.8 IU/L, 20.6 ± 14.6 IU/L, and 1.15 ± 0.39, respectively (Table 2). The percentages of AST ≥ 30 IU/L (30.8%) and ALT ≥ 30 IU/L (59.4%) in those with NAFLD were significantly higher than in non-drinkers without fatty liver (7.6% for AST ≥ 30 IU/L, *p* < 0.0001 and 13.4% for ALT ≥ 30 IU/L, *p* < 0.0001). The percentage of AST/ALT > 1.0 in those with NAFLD (14.8%) was significantly lower than in non-drinkers without fatty liver (60.0%, *p* < 0.0001) (Table 2). Scatter plots of ALT and AST distributions showed that the NAFLD population has higher ALT values over AST values than the non-NAFLD population (Fig. 4).

**Table 2:** AST, ALT, and AST/ALT ratio distribution among Hiroshima residents who had abdominal ultrasonography during check-up

	N	AST (IU/L)				ALT (IU/L)				AST/ALT (AAR)			
		Mean ± SD	Median	Min–max	AST ≥ 30 IU/L (%)	Mean ± SD	Median	Min–max	ALT ≥ 30 IU/L (%)	Mean ± SD	Median	Min–max	ALT/ALT > 1.0 (%)
Total population who received ultrasonography	5999	23.4 ± 12.0	21	8–363	15.4%	26.2 ± 20.6	20	2–503	26.5%	1.06 ± 0.39	1.0	0.26–7	47.8%
Non-drinkers without fatty liver	3781	20.9 ± 9.8*	19	8–363	7.6%^#^	20.6 ± 14.6*	18	2–503	13.4%^#^	1.15 ± 0.39*	1.1	0.26–7	60.0%^#^
Non-drinkers with fatty liver (NAFLD)	1399	28.0 ± 13.6*	25	11–167	30.8%^#^	40.7 ± 26.2*	33	7–291	59.4%^#^	0.78 ± 0.26*	0.73	0.34–2.47	14.8%^#^
*p* value		< 0.0001*			< 0.0001^#^	< 0.0001*			< 0.0001^#^	< 0.0001*			< 0.0001^#^

*AST* alanine aminotransferase, *ALT* asparate aminotransferase, *AAR* AST/ALT ratio, *SD* standard deviation, *NAFLD* non-alcoholic fatty liver disease;

*p value of the Wilcoxon test comparing the AST and ALT levels and AST/ALT ratios in non-drinkers without fatty liver and non-drinkers with fatty liver (NAFLD)

^#^*p* value of the Chi-square test comparing the percentage of AST ≥ 30 IU/L, ALT ≥ 30 IU/L, and ALT/ALT > 1.0 in non-drinkers without fatty liver and non-drinkers with fatty liver (NAFLD)

## Discussion

This is the first report of the distribution of FIB-4 index in a large-scale general population who received ultrasonography at health checkups. Two main new findings were identified. First, when using the recommended FIB-4 index cutoff of 1.3, the percentage of people who met the criteria for referral to hepatologists from health checkups facilities was quite different by age group. Second, the FIB4-index in non-drinkers without fatty liver was higher than in the NAFLD population.

Although it has been reported that the FIB4-index is age-dependent in patients with liver diseases [10, 11], it was also higher in the elderly in the total population who received ultrasonography at health checkups. FIB-4 index ≥ 2.67, which is the criterion for high risk of liver fibrosis [3], was found in 3.8% of those aged 60–69 years and 16.4% of those aged ≥ 70 years, suggesting that age has a significant impact on FIB-4 index in the population who received ultrasonography at health checkups. FIB-4 index was principally developed by analyzing patients with an average age of 40 ± 7 years [12]. It has been reported that the appropriate cutoff for FIB-4 index may vary by age because its formula includes this parameter [13, 14]. This study revealed that high FIB-4 index values were found predominantly in the elderly and that the effect of age is not negligible in those who received ultrasonography at health checkups. Therefore, it may be unsuitable as primary screening for liver fibrosis in the general population even though it can be easily calculated using routine clinical indices. The guidelines [3, 15] indicated that FIB-4 index should be used for patients with diagnosed liver disease. By using the recommended FIB-4 index cutoff of 1.3 [3, 7, 16], this study presented the percentage of people with fatty liver who can be reassured that advanced fibrosis is ruled out: 96.4% in under 50 s, 73.7% in 50 s, 41.9% in 60 s, and 11.9% over 70 years old. However, the percentage of people who met the criteria for referral to hepatologists was unexpectedly high in the elderly (58.1% in the 60–69 age group and 88.1% in those ≥ 70 years). For the elderly, it would be better to re-establish the cutoff value of FIB-4 index in order to reduce unnecessary referrals. Because of its high negative predictive value (NPV), the FIB-4 index is regarded to be beneficial in excluding advanced hepatic fibrosis, but its positive predictive value (PPV) is not as high in detecting advanced fibrosis [8]. In a primary care referral pathway, there is a report recommending the use of a Fib-4 index below 1.0 as the cutoff to distinguish between F0 and F1-4, but it should be noted that the reference data were from young and healthy donors [17] Based on the results of our study, it is clear that the FIB4-index cutoff value should be set by age group. As for sex, no significant difference was found in this study. Regarding other factors, it has been pointed out that obesity and type 2 diabetes may lower the FIB4-index [18–21]. Thrombocytosis due to chronic inflammation may also influence the value of FIB4-index. On the other hand, when platelets decrease due to the influence of drugs or other reasons, FIB4-index value is suspected to increase.

We found that FIB-4 index was significantly lower in those with NAFLD than in the non-drinkers without fatty liver population in each age group, which indicates that NAFLD has a lower FIB4 value than non-NAFLD regardless of age. To explore the reason, AST/ALT distributions were examined since FIB-4 index formula contains AST in the numerator and ALT in the denominator. Non-drinkers without fatty liver had higher AST levels relative to ALT levels (60% with AST/ALT > 1.0), with only 7.6% and 13.4% having increased AST or ALT ≥ 30 IU/L, respectively. In contrast, in the NAFLD population, only 14.8% had higher AST levels relative to ALT levels, with 30.8% and 59.4% showing AST or ALT values of 30 IU/L or higher. This contrast in ALT and AST distributions in the two groups may have resulted in a higher FIB-4 index in the non-drinkers without fatty liver than in the NAFLD population because of the nature of FIB-4 index formula.

In this study, 69.2% and 40.6% of NAFLD patients had AST and ALT levels within the normal ranges. However, increased AST and ALT levels, even within the normal ranges, and decreased AST/ALT ratios were reported to be associated with an increased risk of metabolic abnormalities and metabolic syndrome [22]. AST is distributed in the heart, lungs, liver, kidneys, muscles, and erythrocytes and has low liver specificity. ALT is located in abundance in the cytoplasm of hepatocytes and has high liver specificity. Liver disorders cause increased serum AST and ALT levels. However, due to the difference in half-life between AST and ALT, patients with alcoholic liver disease or cirrhosis have AST > ALT, whereas patients with chronic hepatitis or fatty liver have ALT > AST [23, 24]. Although increased ALT level is considered a surrogate marker for NAFLD [25–27], ALT level is known not to correlate with histological findings and disease severity of NAFLD [28, 29]. FIB-4 index is a useful marker of liver fibrosis for patients with any liver disease because its formula contains AST in the numerator and ALT in the denominator. However, FIB-4 index might not be appropriate for the healthy population because it also has higher AST levels relative to ALT levels.

There are some limitations in this population-based study. First, the result of liver biopsy is absent. Thereby, verification of FIB-4 index results against histological findings could not be done. Although many hospital-based studies suggest that FIB-4 index is a highly accurate liver fibrosis marker [6, 15, 29–31], its accuracy in the general population, especially in the elderly, needs to be verified in the future. Second, the study subjects were limited to those who have undergone ultrasonography at health checkups. As people with liver disease tend to undergo ultrasonography, the control group without NAFLD could include people with other liver diseases, thus altering the AST/ALT ratio. However, since it is a population from health checkups, the frequency of including patients with serious liver disease is expected to be low. Third, the diagnosis of fatty liver was based on ultrasonography which is operator-dependent. Other non-invasive modalities such as transient elastography and magnetic resonance elastography were not commonly used during health checkups, and existing large-scale data could not be obtained for this study. It is desirable that transient elastography spread to health checkups facilities in the future, but at present, ultrasonography is common and conventional. Forth, since this study targeted only Japanese people, extrapolation to other ethnic groups should be cautious, and comparative studies are needed to confirm our findings. Fifth, it has been pointed out that obesity and diabetes affect the value of the FIB-4 index [18–21], but this study did not evaluate it.

## Conclusions

Fatty liver is one of the most frequent findings in abdominal ultrasonography at health checkups, and it is critical to find cases with advanced fibrosis. FIB-4 index is considered a useful marker for evaluating liver fibrosis. However, since in the NAFLD population, 58.1% of those in their 60 s and 88.1% of those ≥ 70 years met the criteria for referral to hepatologists (FIB-4 index ≥ 1.3), it would be better to re-establish the cutoff value for the elderly to avoid over-referral. In addition, for the general population, due to the impact of age and characteristics of AST/ALT ratios, there is no prospect of using FIB-4 index for primary screening for liver fibrosis even though it can be easily calculated using routine clinical indices. It is desired to develop a non-invasive method for picking up cases with advanced fibrosis latent in the general population.

## Supplementary Information

**Additional file 1. Fig. S1.** Proportion of age group in each FIB-4 Index value in residents who underwent ultrasonography at health checkups by gender.

**Additional file 2. Table S1.** Comparison of FIB-4 index between non-drinkers without fatty liver and non-drinkers with fatty liver (NAFLD).

## Data Availability

The datasets used and/or analyzed in the current study are available from the corresponding author on reasonable request.

## Abbreviations

NAFLD: Non-alcoholic fatty liver disease
NAFL: Non-alcoholic fatty liver
NASH: Non-alcoholic steatohepatitis
FIB-4: Fibrosis-4
AST: Aspartate aminotransferase
ALT: Alanine aminotransferase
HBV: Hepatitis B virus
HCV: Hepatitis C virus
SD: Standard deviation
AAR: AST/ALT ratio
