# Supplementary material for: Distribution of FIB-4 index in the general population: analysis of 75,666 residents who underwent health checkups
Source: BMC Gastroenterol. 2022 May 13;22:241. doi: 10.1186/s12876-022-02290-1 (PMC9101936; doi:10.1186/s12876-022-02290-1)
Supplement: Supplementary file 1 — Additional file 1. Fig. S1. Proportion of age group in each FIB-4 Index value in residents who underwent ultrasonography at health checkups by gender. [file 12876_2022_2290_MOESM1_ESM.pdf]

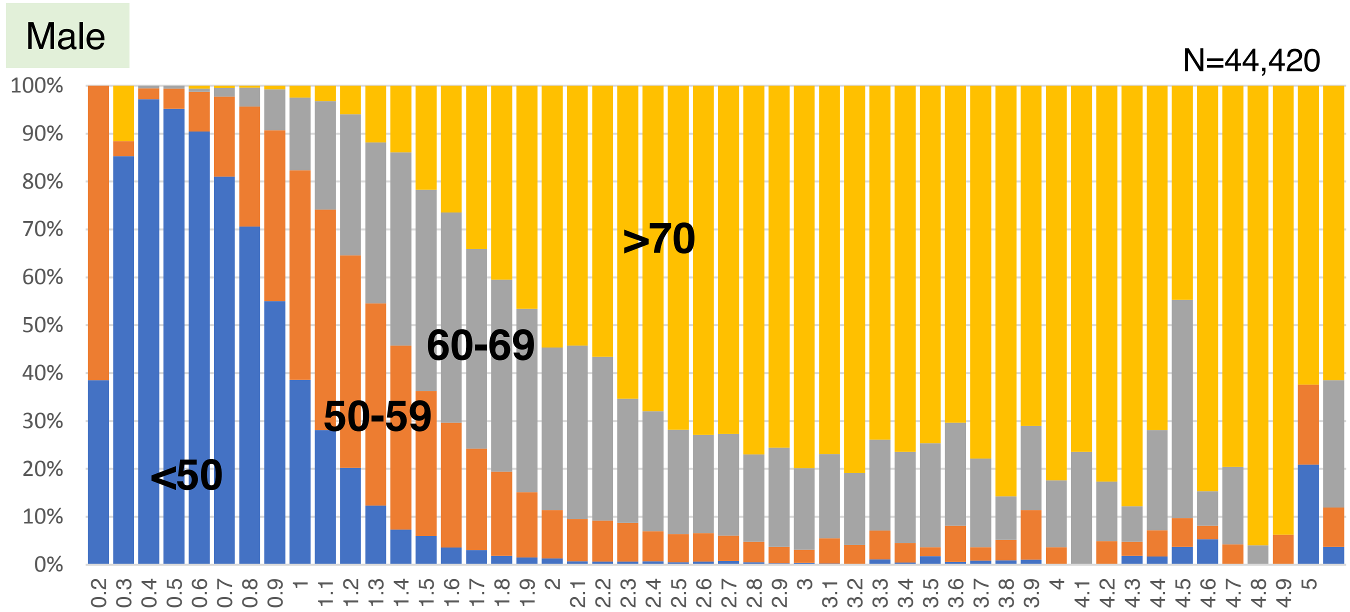

Age	N	FIB-4 index Mean ± SD
<50	19,426	0.81±0.32
50-59	12,165	1.23±0.47
60-69	9,578	1.63±0.67
≥70	3,251	2.18±0.76
**Total**	**44,420**	**1.21±0.65**

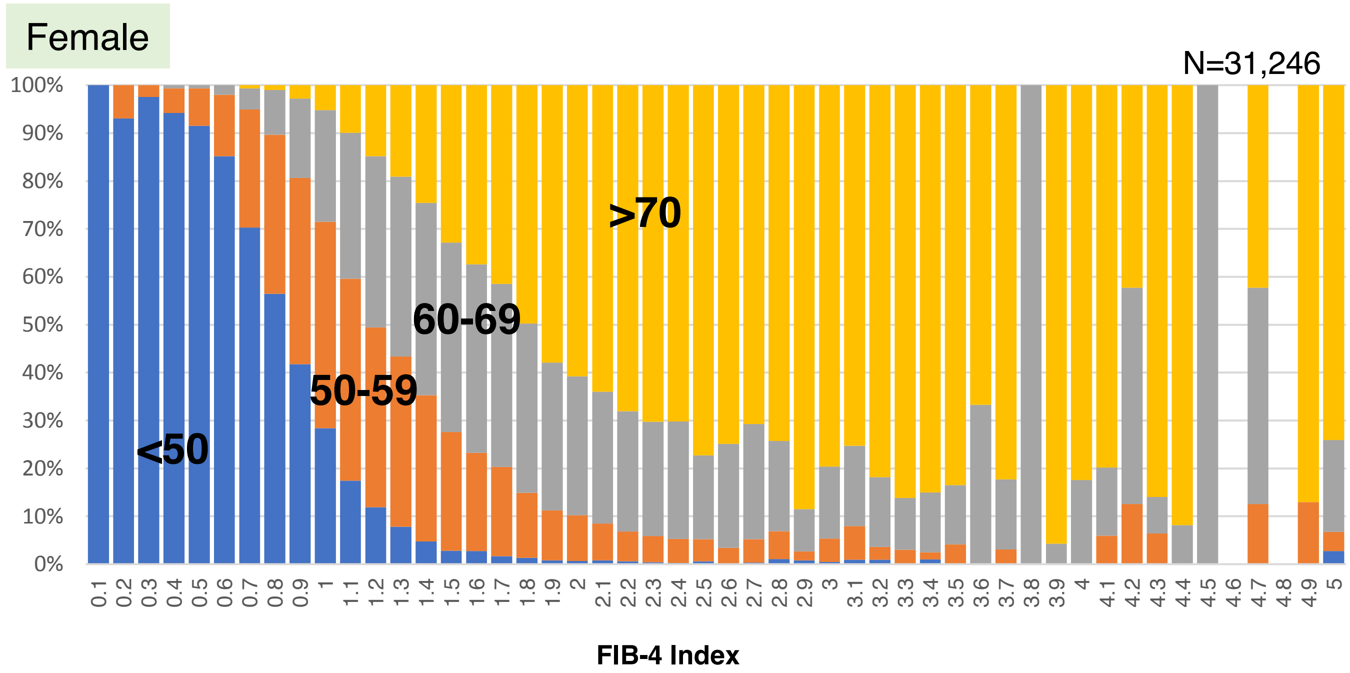

Age	N	FIB-4 index Mean ± SD
<50	12,677	0.82±0.29
50-59	8,703	1.23±0.39
60-69	7,276	1.55±0.65
≥70	2,590	2.00±0.72
**Total**	**31,246**	**1.20±0.60**

Supplementary Figure 1. Proportion of age group in each FIB-4 Index value in residents who underwent ultrasonography at health checkups by gender
