# Supplementary material for: Distribution of FIB-4 index in the general population: analysis of 75,666 residents who underwent health checkups
Source: BMC Gastroenterol. 2022 May 13;22:241. doi: 10.1186/s12876-022-02290-1 (PMC9101936; doi:10.1186/s12876-022-02290-1)
Supplement: Supplementary file 2 — Additional file 2. Table S1. Comparison of FIB-4 index between non-drinkers without fatty liver and non-drinkers with fatty liver (NAFLD). [file 12876_2022_2290_MOESM2_ESM.docx]

| **Age**  **(years)** | **FIB-4 index** | | | | | **Wilcoxon test** |
| --- | --- | --- | --- | --- | --- | --- |
|  | **Non-drinkers without fatty liver（N=47,222）** | |  | **Non-drinkers with fatty liver (NAFLD) （N=17,968）** | |  |
|  | **N** | **Mean ± SD** |  | **N** | **Mean ± SD** |  |
| <50 | 20,133 | 0.82 ± 0.28 |  | 6,934 | 0.75 ± 0.31 | p<0.0001 |
| 50–59 | 11,946 | 1.24 ± 0.40 |  | 5,559 | 1.13 ± 0.38 | p<0.0001 |
| 60–69 | 10,649 | 1.61 ± 0.63 |  | 4,350 | 1.48 ± 0.66 | p<0.0001 |
| ≥70 | 4,494 | 2.13 ± 0.75 |  | 1,125 | 1.92 ± 0.66 | p<0.0001 |
| Overall | 47,222 | 1.23 ± 0.63 |  | 17,968 | 1.12 ± 0.58 | p<0.0001 |

Supplementary Table 1. Comparison of FIB-4 index between non-drinkers without fatty liver and non-drinkers with fatty liver (NAFLD)
